# Protocolized oxytocin infusion for elective cesarean delivery: a retrospective before-and-after study

**DOI:** 10.1007/s00540-024-03329-1

**Published:** 2024-03-22

**Authors:** Azusa Nagai, Yuki Shiko, Shohei Noguchi, Yusuke Ikeda, Yohei Kawasaki, Yusuke Mazda

**Affiliations:** 1 Department of Obstetric Anesthesiology, Center for Maternal-Fetal and Neonatal Medicine, Saitama Medical Center, Saitama Medical University, 1981 Kamoda, Kawagoe, Saitama 350-8550 Japan; 2 Department of Anesthesiology, Saitama Medical Center, Saitama Medical University, Kawagoe, Japan; 3 Biostatistics Section, Clinical Research Center, Chiba University Hospital, Chiba University, Chiba, Japan; 4 Faculty of Nursing, Japanese Red Cross College of Nursing, Tokyo, Japan

**Keywords:** Anesthesia, Cesarean delivery, Oxytocin, Protocol, Postpartum hemorrhage

## Abstract

**Purpose:**

To elucidate the clinical impact of the novel oxytocin protocol using a syringe pump with a stratified dose compared with the conventional practice of putting oxytocin into the bag.

**Methods:**

This is a retrospective cohort study. We collected the data of the patients who underwent elective cesarean delivery under neuraxial anesthesia between June 2019 and May 2020. The patients were allocated to two groups according to oxytocin administration methods; the control group (the attending anesthesiologist put oxytocin 5–10 units in the infusion bag and adjusted manually after childbirth) and the protocol group (the oxytocin protocol gave oxytocin bolus 1 or 3 units depending on the PPH risk, followed by 5 or 10 unit h^−1^ via a syringe pump). We compared the total amount of oxytocin within 24 h postpartum, estimated blood loss, and adverse clinical events within 24 h postpartum between the two groups.

**Results:**

During the study period, 262 parturients were included. Oxytocin doses of intraoperative and postoperative were significantly lower in the protocol group (9.7 vs. 11.7 units, intraoperative, 15.9 vs. 18 units, postoperative). The subgroup analyses showed that the impact was more remarkable in the low PPH risk than in the high PPH risk. The multivariate linear regression analyses also confirmed the difference. The groups had no significant difference in blood loss, requirement of additional uterotonics, and other adverse events.

**Conclusions:**

Our oxytocin infusion protocol significantly reduced oxytocin requirements in elective cesarean delivery under neuraxial anesthesia without increasing blood loss. However, we could not find other clinical benefits of the novel protocol.

**Supplementary Information:**

The online version contains supplementary material available at 10.1007/s00540-024-03329-1.

## Introduction

Oxytocin is a gold standard for preventing postpartum hemorrhage (PPH) in childbirth [1]. In cesarean delivery (CD), the risk of PPH is higher than vaginal delivery [2, 3]; thus, immediate administration of oxytocin is recommended. However, there are multiple administration methods, depending on local practice and cultural differences [4, 5]. In general, many anesthesiologists administer oxytocin by adding it into an intravenous fluid bag, which is common practice in Japan [6]. The method is easily implemented but occasionally fluctuates the dose by changing the infusion speed, leading to unstable uterotonic effects. Furthermore, this practice may lead to an oxytocin overdose if PPH develops and fluid resuscitation is instituted with this intravenous bag containing oxytocin. Oxytocin overdose leads to various adverse effects, such as hypotension, tachycardia, nausea, ST-segment change on electrocardiogram (ECG) [7], or cardiac arrest. To balance the clinical benefit and adverse event, we should ideally give oxytocin and volume in a separate infusion route.

The appropriate doses of oxytocin, 0.35 IU (95% CI 0.18–0.52 IU) for elective and 2.99 IU (95%CI 2.32–3.67) for intrapartum cesarean delivery, have been elucidated by obstetric anesthesiologists for over two decades [8, 9]. Of note, the oxytocin requirement for adequate uterine contraction varies by oxytocin exposure [10]. Moreover, the doses found by anesthesiologists are much lower than the recommended dose by other societies [11, 12]. In 2019, an international group of obstetric anesthesiologists published the consensus on uterotonics in CD [5]. They recommend stratified doses by the clinical situation, low dose for elective and high dose for intrapartum CD. However, the clinical impact of implementing the stratification for PPH prevention in CD is still unknown. We hypothesized that if we use oxytocin in a stratified manner, the total dose of oxytocin and even adverse events related to oxytocin might decrease.

In our institute, we used to put uterotonics in the bag and adjust the infusion speed manually without using any pumps, preventing PPH in CD. The attending anesthesiologists had chosen their oxytocin dose at their discretion and gave second-line uterotonics at the obstetricians’ requests. As a result, parturients might be often overexposed to oxytocin during surgery and develop uterotonics-related adverse events. To overcome the issue, we have organized a small Quality Improvement team and developed a protocol of meticulously infusing oxytocin via a syringe pump. In this retrospective study, we aimed to assess the clinical impact of the oxytocin protocol, which might decrease both intraoperative oxytocin dose and adverse events without increasing PPH.

## Materials and methods

After obtaining approval from the Institutional Review Board at the Saitama Medical Center, Saitama Medical University (#2593, Kawagoe, Japan), we conducted this before-and-after study. Due to the manner of the retrospective study design without any controlled population, we waived to obtain informed consent from each participant. All medical and anesthesia records were reviewed using an electronic medical chart, and data on maternal characteristics, the indication of CD, and intra- and post-operative status were extracted (Supplemental table). We included pregnant women aged over 17 years, American Society of Anesthesiology Physical Status of II or III, non-laboring, elective CD with neuraxial anesthesia between June 2019 and May 2020. Our exclusion criteria were a history of oxytocin allergy, required general anesthesia conversion at any time of surgery, and non-elective CD.

All patients with elective CD were fasted for over 8 h before surgery. Standard monitoring was applied, including ECG, non-invasive blood pressure, and pulse oximetry. Blood pressure was recorded every minute from induction of anesthesia to delivery of the fetus, followed by every 2.5 min until the end of surgery. After inserting an 18- or 20-gauge peripheral venous line on her arm, 10 mg of metoclopramide was given routinely. Spinal anesthesia was performed at L2/3 or L3/4 interspace using a 27-gauge Whitacre needle or a combined spinal-epidural needle in the sitting or lateral position at the discretion of anesthesiologists. All parturients were intrathecally received hyperbaric bupivacaine 12 mg with fentanyl 10 µg and preservative-free morphine 150 µg. After intrathecal injection, the patient was positioned supine and applied left uterine displacement using a wedge under the right hip until delivery. To avoid maternal hypotension, the co-loading of colloid 0.5 to 1 litter (Volven®; Otsuka Pharmaceutical Factory, Tokushima, Japan) and prophylactic norepinephrine infusion of 2.5 to 5.0 µg/min, which tapered off after delivery, were applied to all women.

Our classic management of uterotonics was as follows; initially putting 5 to 10 units of oxytocin in a primary 500-mL fluid bag immediately after the childbirth; the roller clamp was widely opened for one to two minutes and then adjusted by the anesthesiologist; if the team clinically detected uterine atony, additional oxytocin 5 to 10 units put in the bag or intravenous ergometrine with slow injection. A secondary bag of fluid was rarely used unless putting another intravenous line. At the end of the surgery, the fluid bag was manually adjusted to maintain approximately 80 milliunits/h.

**Fig. 1:**
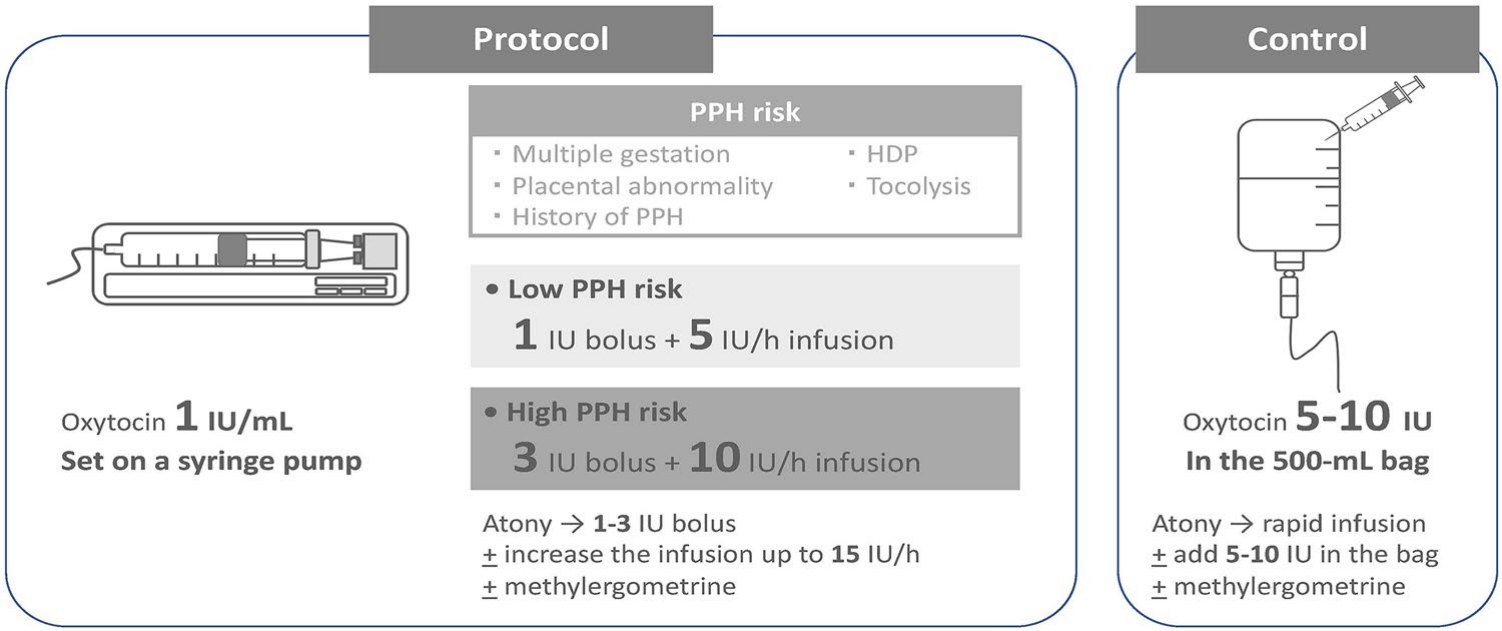
Oxytocin protocol at the Saitama Medical Center, Saitama Medical University. *PPH* postpartum hemorrhage, *HDP* hypertensive disorders of pregnancy

For our uterotonics protocol, we stratified the dose of oxytocin by the PPH risk (multiple gestations, placental-position abnormality, history of previous PPH, hypertensive disorders of pregnancy, tocolysis within 24 h; we treated it as high risk if even one of several risk factors was present). Fifteen units of oxytocin were diluted in 15 mL with the final concentration of 1 unit/mL in a 20-mL syringe. Oxytocin infusion was initiated immediately after childbirth via a syringe pump. In the no PPH risk, we gave an initial bolus of 1 unit followed by 5 unit/h, and in the PPH risk, an initial bolus of 3 units followed by 10 unit/h (Fig. 1). If the team clinically detected uterine atony, we gave further one to three units of oxytocin and increased the infusion speed up to 15 unit/h. When the increased oxytocin did not work in three to five minutes, 0.2 mg of intravenous ergometrine was given in the same manner as our classic management. At the end of the surgery, the fluid bag was switched to a new bag with oxytocin of 10 U/L, and the roller clamp was adjusted manually to maintain approximately 80 milliunits/h.

The following practices were the same between conventional group and updated group with protocol. Tranexamic acid was given by the request of the obstetric team during and after surgery. After the surgery, the obstetric team continued oxytocin infusion for 12 to 18 h, and their clinical decision gave further ergometrine. The vaginal bleeding was counted by the nurses or midwives at least every 4 h until 24 h postpartum. In addition, they assessed the parturients every hour for clinical adverse events. All parturients were monitored through pulse oximetry for 24 h, and if the parturients had tocolysis before 24 h of delivery, we also monitored ECG for 24 h. On the following day of surgery, her hemoglobin and hematocrit were routinely assessed by the complete blood count.

Our primary outcome was the intraoperative dose of oxytocin extracted from the anesthetic records. The oxytocin dose through the fluid bag was calculated by the remaining amount of the fluid. The secondary outcomes included the amount of blood loss, maternal adverse events, such as nausea and vomiting, hypotension, tachycardia, cardiac arrest, any ECG change noted in the anesthesia chart or medical records, the total amount of synthesized oxytocin in 24 h postpartum, additional uterotonics requirement, and hemoglobin change before and after the surgery.

### Sample size and statistical analysis

The sample size was justified by the primary outcome and the hypothesis. The preliminary data from our internal analysis revealed that our classic management required intraoperative oxytocin 11.3 ± 4.5 [mean ± standard deviation (SD)]. It was our hypothesis that implementing the oxytocin protocol group (protocol group) would decrease by at least 20% in the primary outcome compared with our classic management (control group). We estimated that a total of 166 patients (83 for each group) would be required to achieve 90% power to detect the 20% reduction, assuming a significant level of 0.05. We generally have 250 elective cesarean deliveries annually, and our protocol was implemented in December 2019; however, due to the pandemic situation, we could not presume the elective CD during the study period before conducting the protocol. Thus, we decided to include the data of participants until May 2020.

The primary outcome was compared between the two groups using the Student’s *t*-test. We also conducted multivariate linear regression analyses to mitigate potential biases inherent in a retrospective observational study by controlling for cofounding factors. In the multivariate linear regression analyses, the following covariates were used to adjust for oxytocin protocol and confounders: PPH risk, maternal age, term or preterm birth, estimated blood loss, intraoperative methylergometrine, preoperative values of hemoglobin, activated partial thromboplastin time and prothrombin time. The secondary outcomes were compared between the two groups using the Chi-square test or Fisher’s exact test as appropriate for categorical variables and the Student’s *t *test for continuous variables. Subgroup analyses of the PPH risk were also conducted to compare the groups. Data were analyzed using Stata/MP for Mac version 13.0 (Stata Corporation, College Station, TX, USA), and a two-sided *p*-value of 0.05 was used to determine the statistical significance.

## Results

**Fig. 2:**
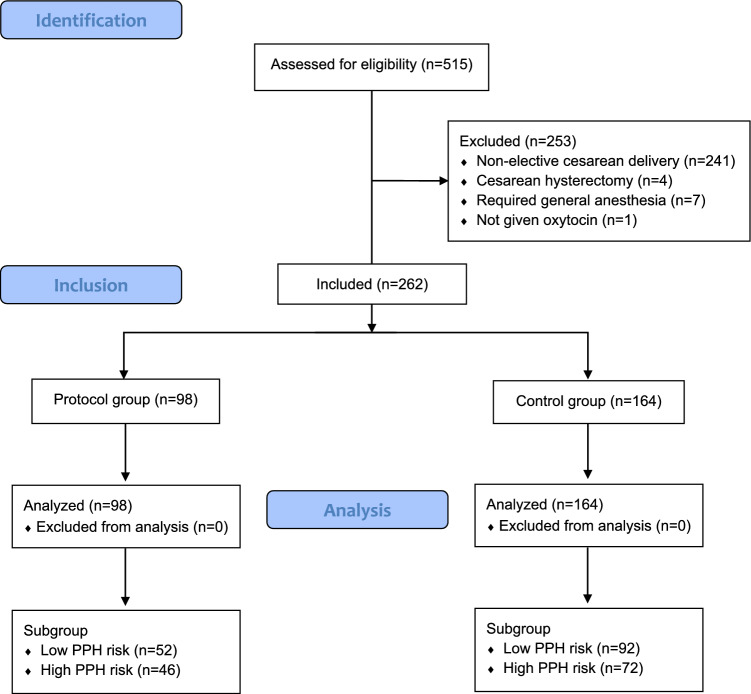
STROBE diagram. *STROBE* Strengthening the Reporting of Observational Studies in Epidemiology Statement, *PPH* postpartum hemorrhage

Two hundred and seventy out of 515 parturients were eligible for the study, but one did not receive oxytocin, and seven required general anesthesia; therefore, 262 women were included in the analysis (Fig. 2). Apart from the proportion of nulliparous, the duration of surgery and preoperative activated partial thromboplastin time, there was no difference between the groups (Table 1). The intraoperative, postoperative, and total amount of oxytocin was significantly lower in the protocol group, but the reduction of total oxytocin dose in 24-h postpartum was − 4.18 units [95% CI − 5.57 to − 2.79] (Table 2). Multivariate linear regression analysis showed the same result (adjusted reduction of total oxytocin dose in 24-h postpartum was − 3.91 units [95% CI − 5.22 to − 2.60] (Table 3). We could not find further improvement in additional uterotonics, reduction of postpartum hemorrhage, an additional procedure for PPH, and adverse events in the protocol group (Table 2). There were no parturients who received tranexamic acid during and after cesarean delivery.

**Table 1:** Patient characteristics

	Protocol (*n* = 98)		Control (*n* = 164)		*p* value
	Mean/*N*	SD/%	Mean/*N*	SD/%	
Age, year	34.7	6.1	35	5	0.64
Height, cm	158	5.7	157.3	9.7	0.5
Weight, kg	63.8	11.8	65.2	11.9	0.37
BMI, kg/m^−2^	25.6	4.7	27.1	12.3	0.26
Nulliparous, *n* (%)	52	53%	65	40%	0.03
Gestational age, week	36.8	0.9	36.9	0.8	0.72
Preterm delivery, *n* (%)	35	36%	46	28%	0.19
Fetal growth restriction, *n* (%)	10	10%	9	5%	0.15
ASA-PS, 2/3	94/4		162/2		0.13
Hypertensive disorders of pregnancy, *n* (%)	8	8%	11	7%	0.66
Diabetes, T1DM/T2DM/GDM, *n*	1/2/9		2/1/16		0.77
Coagulopathy, *n* (%)	2	2%	0	0%	0.07
Indication, *n* (%)					0.46
Previous	30	31%	68	41%	
Breech presentation	14	14%	16	10%	
Multiple gestation	26	27%	37	23%	
Placental abnormality	14	14%	19	12%	
Uterine scar	6	6%	14	9%	
Others	8	8%	10	6%	
Surgical time, min	60.5	16.4	69	21	< 0.01
Type of anesthesia, *n* (%)					0.52
Spinal	48	49%	77	47%	
CSEA	47	48%	85	52%	
Epidural	3	3%	2	1%	
PPH risk	46	47%	72	44%	0.63
Preoperative coagulation test					
Platelet count, × 10^3^/µL	221	64	223	61	0.83
APTT, s	28.0	3.1	26.8	2.5	< 0.01
PT, s	11.1	1.1	11.1	0.6	0.86

*SD* standard deviation, *BMI* body mass index, *ASA-PS* American Society of Anesthesiologists physical status, *T1DM* type 1 diabetes mellitus, *T2DM* type 2 diabetes mellitus, *GDM* gestational diabetes mellitus, *CSEA* combined spinal epidural anesthesia, *PPH* postpartum hemorrhage, *APTT* activated partial thromboplastin time, *PT* prothrombin time

**Table 2:** Uterotonics and postpartum hemorrhage

	Protocol (*n* = 98)		Control (*n* = 164)		*p* value	MD/uOR	95% CI
	Mean/*N*	SD/%	Mean/*N*	SD/%			
Oxytocin, IU							
Intraoperative	9.7	4.5	11.7	3.8	< 0.01	− 1.98	− 2.97 to − 0.99
Postoperative	15.9	2.4	18	3.7	< 0.01	− 1.93	− 2.74 to − 1.13
24-h postpartum (total)	25.5	5.2	29.7	5.7	< 0.01	− 3.91	− 5.22 to − 2.60
Methylergometrine, *n* (%)							
Intraoperative	46	47%	84	51%	0.45	0.84	0.51 to 1.39
Postoperative	30	31%	41	25%	0.32	1.32	0.76 to 2.31
24-h postpartum (total)	52	53%	93	57%	0.57	0.86	0.52 to 1.43
Hemorrhage, mL							
Intraoperative	1270	638	1340	604	0.38	70	− 85 to 225
Postoperative	187	135	189	173	0.9	1.9	− 40 to 44
24-h postpartum (total)	1445	671	1509	665	0.46	64	− 104 to 231
Transfusion, *n* (%)	4	4%	8	5%	0.09	0.83	0.24 to 2.83
Hemoglobin							
Preoperative, g/dL	11.3	0.9	11.3	1.0	0.74	− 0.04	− 0.28 to 0.20
Postoperative, g/dL	11.0	1.3	11.0	1.6	0.84	− 0.04	− 0.41 to 0.33
Changes, %	− 2.4	8.5	− 2.3	12	0.94	0.09	− 2.6 to 2.8
Intrauterine tamponade	13	13%	30	18%	0.29	0.68	0.34 to 1.38
Hypotension after delivery, *n* (%)							
Intraoperative	28	29%	44	27%	0.76	1.09	0.62 to 1.91
Postoperative	17	17%	42	26%	0.08	0.61	0.32 to 1.14
Notable ECG changes, *n* (%)							
Intraoperative	3	3%	4	2%	0.76	1.26	0.28 to 5.77
Postoperative	0	0%	7	4%	0.03	N/A	–
Nausea and vomiting, *n* (%)							
Intraoperative	17	17%	20	12%	0.25	1.51	0.75 to 3.05
Postoperative	25	26%	50	30%	0.39	0.78	0.44 to 1.37

*SD* standard deviation, *MD* mean difference, *uOR* unadjusted odds ratio, *CI* confidence interval, *IU* international unit

**Table 3:** Multivariate linear regression analyses; oxytocin dose

	Protocol	Control	Mean difference	95% CI	*p* value
Overall, IU					
Intraoperative	9.69	11.66	− 1.98	− 2.97 to − 0.99	< 0.01
Postoperative	16.21	18.15	− 1.93	− 2.74 to − 1.13	< 0.01
24-h postpartum (total)	25.9	29.81	− 3.91	− 5.22 to − 2.60	< 0.01
PPH low risk, IU					
Intraoperative	8.1	11.7	− 3.6	− 5.0 to − 2.2	< 0.01
Postoperative	15.5	16.7	− 1.1	− 2.2 to − 0.1	0.03
24-h postpartum (total)	23.6	28.3	− 4.7	− 6.5 to − 3.0	< 0.01
PPH high risk, IU					
Intraoperative	11.5	11.8	− 0.3	− 1.7 to 1.1	0.68
Postoperative	16.3	19.2	− 2.9	− 4.3 to − 1.6	< 0.01
24-h postpartum (total)	27.8	31	− 3.2	− 5.2 to − 1.2	< 0.01

*CI* confidence interval, *PPH* postpartum hemorrhage. Covariates were used to adjust for cofounders of maternal age, preterm delivery, estimated blood loss, intraoperative methylergometrine, preoperative hemoglobin/activated partial thromboplastin time/prothrombin time

The secondary analysis of stratifying the preexisting risks of PPH showed that the reduction of intraoperative oxytocin dose was observed in the low PPH risk subgroup; however, we did not find a difference in the high PPH risk subgroup (Table 4). The protocolized oxytocin infusion significantly decreased intraoperative blood loss in the low PPH risk population.

**Table 4:** Subgroup analyses by risks of postpartum hemorrhage

PPH low risk	Protocol (*n* = 52)		Control (*n* = 92)		*p* value	MD/aOR	95% CI
	Mean/*N*	SD/%	Mean/*N*	SD/%			
Oxytocin, IU							
Intraoperative	7.8	4.1	11.8	4	< 0.01	− 3.94	− 5.31 to − 2.56
Postoperative	15.5	2	16.7	3.2	0.01	− 1.26	− 2.23 to − 0.29
24-h postpartum (total)	23.3	4.6	28.5	5.6	< 0.01	− 5.2	− 6.99 to − 3.40
Methylergometrine, *n* (%)							
Intraoperative	19	37%	34	37%	0.96	0.98	0.49 to 1.99
Postoperative	11	21%	14	15%	0.37	1.49	0.62 to 3.59
24-h postpartum (total)	21	40%	38	41%	0.91	0.96	0.48 to 1.92
Hemorrhage, mL							
Intraoperative	937	343	1152	393	< 0.01	− 215	− 344 to − 86
Postoperative	180	146	167	128	0.57	14	− 34 to 62
24-h postpartum (total)	1107	416	1306	413	< 0.01	− 199	− 341 to − 57

PPH High risk	Protocol (*n* = 46)		Control (*n* = 72)		*p* value	MD/aOR	95% CI
	Mean/*N*	SD/%	Mean/*N*	SD/%			
Oxytocin, IU							
Intraoperative	11.7	4.1	11.7	3.7	0.93	0.06	− 1.37 to 1.50
Postoperative	16.3	2.7	19.6	3.8	< 0.01	− 3.28	− 3.28 to − 4.56
24-h postpartum (total)	28	4.7	31.2	5.6	< 0.01	− 3.21	− 5.18 to − 1.25
Methylergometrine, *n* (%)							
Intraoperative	27	59%	50	69%	0.23	0.63	0.29 to 1.35
Postoperative	19	41%	27	38%	0.68	1.17	0.55 to 2.50
24-h postpartum (total)	31	67%	55	76%	0.28	0.64	0.28 to 1.45
Hemorrhage, mL							
Intraoperative	1646	687	1579	732	0.63	67	− 201 to 334
Postoperative	194	122	219	219	0.5	− 25	− 98 to 48
24-h postpartum (total)	1827	701	1768	821	0.69	59	− 231 to 350

*PPH* postpartum hemorrhage, *SD* standard deviation, *MD* mean difference, *aOR* adjusted odds ratio, *CI* confidence interval, *IU* international unit

## Discussion

Our results demonstrated that the protocolized oxytocin infusion in elective cesarean delivery reduced the total amount of oxytocin dose without compromising PPH; however, it did not improve other clinical outcomes, except for postoperative ECG change. The reduction was observed not only during the cesarean delivery but also after delivery. In addition, the subgroup analysis revealed that the intraoperative oxytocin dose was significantly reduced in the population with low risk of PPH, not in the high PPH risk parturients.

The optimal dose of oxytocin has been investigated for over two decades [8, 9]. Recently, the international consensus for preventing PPH during cesarean delivery recommends that clinicians should initiate the oxytocin with the lowest effective dose (a unit bolus for elective, three units of bolus for non-elective) and titrate in a step-by-step manner depending on the clinical situation [5]. As the sensitivity of oxytocin to uterine contraction varies by the endogenous and exogenous exposures before delivery [9, 10], the dose–response curve is not the same. Also, some population requires a higher dose of oxytocin for adequate uterine contraction even in the oxytocin naive status [13, 14]; thus, it is reasonable to initiate a relatively higher oxytocin dose to prevent PPH in the high-risk population. We adopted the stratified protocol for preventing uterine atony in cesarean delivery, which was clinically feasible and plausible.

According to our subgroup analysis, the oxytocin reduction was more significant in the low PPH risk subgroup not in the high PPH risk subgroup. Our primary practice of oxytocin infusion (putting 5 to 10 units of oxytocin into the 500-mL bag and running fast, then slowing it down manually) was standard. Still, the average dose required over 10 units, and the dose was relatively large for the low PPH risk population. Thus, our protocol worked specifically on this group, not the high PPH risk parturients. The currently available evidence allows us to reduce only dose of oxytocin in the low-risk, not reducing even amount of bleeding, but we are still missing tangible evidence to prevent developing PPH. Not only an appropriate dose of uterotonics but also additional pharmacological strategies, such as early second-line uterotonics or supplemental intravenous calcium chloride [15, 16], would be considered. It is conceivable that the PPH high-risk group may encompass states of oxytocin desensitization, placental malposition such as previa placenta leading less hemostasis by uterine contraction, or preexisting uterine stretching as seen in multiple pregnancies, which may inherently render uterotonic administration less effective in preventing PPH. Specifically, the ED90 of oxytocin required to achieve adequate uterine contraction in twin pregnancies has been reported 3.41 to 4.38 IU [17], suggesting that a one-size-fits-all oxytocin regimen may not suffice for the PPH high-risk population. Instead, additional strategies such as routine administration of tranexamic acid and improved surgical techniques for uterine suturing may be necessary. Therefore, our findings suggest that the significant reduction in oxytocin dosage as a result of protocol improvement was only observed in the PPH low-risk group, indicating that different strategies may be required to manage PPH effectively in high-risk groups.

The dose reduction of oxytocin in our study was relatively small, and some may argue that only less than a five-unit reduction might not be clinically significant [18]. However, the oxytocin supply chain has not been stable worldwide because of temperature instability. Indeed, supply disruptions in distributing oxytocin to obstetric facilities happened in many countries at different time points (Japan in 2011 immediately after the earthquake, Ghana [19] in 2015, and Canada [20] in 2019). Carbetocin would be the best alternative for preventing PPH during cesarean delivery, but its availability is still limited. Given oxytocin is an essential medication in obstetrics for treating and preventing PPH, it would be life-threatening in the lack of a steady supply chain. A reading cause of maternal mortality and morbidity, even a slight reduction of oxytocin would be clinically crucial from a socio-medical point of view.

In our institute, as the obstetric team was responsible for postpartum oxytocin infusion in the postpartum ward, we did not intervene in their management. Despite that, oxytocin dose after surgery up to 24-h postpartum was remarkably reduced. We speculated that an appropriate amount of oxytocin would not interfere endogenous secretion of oxytocin from the maternal pituitary gland. Excessive oxytocin might hinder natural oxytocin secretion because synthetic oxytocin does not pass the brain–blood barrier, and peripheral oxytocin affects the regulation of oxytocin secretion [21]. Intrapartum synthetic oxytocin infusion would interact with breastfeeding and mother-to-infant bonding [22]; it would be logical that unnecessary oxytocin negatively impacted PPH [23]. On the other hand, some deny the clinical relationship between synthetic oxytocin and mother-to-infant bonding [24]. To elucidate our insight, further investigation is needed.

Our study has several limitations. First, although we developed a relatively simple protocol, the compliance rate was 68%, which was 77% in the low PPH risk subgroup and 54% in the high PPH risk subgroup. The issue might arise because our stratification had several controversial points. For instance, we included hypertensive disorders of pregnancy as a PPH risk. Still, some clinicians hesitate to give three units of oxytocin bolus to hypertensive women due to potential hemodynamic instability. In addition, the previa placenta is at a considerable risk of PPH, but oxytocin is not the sole factor in preventing PPH in the etiology. However, three or more stratification will make the protocol complex, and it will not work feasibly. The extreme meticulousness would lose acceptance from the clinicians. Second, our hospital is a tertiary perinatal center only having high-risk pregnancies; thus, our study population would not represent all pregnant women. As the oxytocin reduction was more significant in the low PPH population, our results would be addressed in the low-risk pregnancies. Additionally, our study's retrospective design imposed constraints due to the limited number of cases, preventing a comprehensive analysis that accounted for confounding factors related to uterine atony and postpartum hemorrhage, such as the history of cesarean delivery and postpartum hemorrhage, diabetes mellitus, macrosomia, platelet count, and fibrinogen levels. This limitation led to potential biases in our results due to inadequate adjustment for these factors. The comparability of our two study groups is also questionable, as the baseline characteristic p-values were not significantly different in most instances, which complicates the assertation of equivalent groups. Furthermore, in this study, the sample size was designed based on a t test of the primary outcome, as multiple regression analysis was not used as the primary method of analysis. In addition, the subgroup analyses were of an exploratory nature and were conducted with a smaller sample size; therefore, the results from these analyses should be interpreted with caution. Finally, the assessment of the uterine contraction after delivery was subjective and only made by the obstetricians’ perception. As this study was a part of our quality improvement project, our data should be considered real-world data, which can reflect ubiquitous practices in obstetrics. To overcome these flaws, a further prospective multi-center project involving multidisciplinary professionals would be needed.

## Conclusions

Meticulously infused oxytocin using a syringe pump with risk stratification of PPH significantly reduced the total amount of oxytocin during and after elective cesarean delivery without compromising the PPH.

### Supplementary Information

Below is the link to the electronic supplementary material.

Supplementary file1 (DOCX 16 KB)

## Data Availability

Raw data were generated at Saitama Medical University. Derived data supporting the findings of this study are available from the corresponding author, Yusuke Mazda, on request.
