# Supplementary material for: Protocolized oxytocin infusion for elective cesarean delivery: a retrospective before-and-after study
Source: J Anesth. 2024 Mar 22;38(4):425–33. doi: 10.1007/s00540-024-03329-1 (PMC11284190; doi:10.1007/s00540-024-03329-1)
Supplement: Supplementary file 1 — Supplementary file1 (DOCX 16 KB) [file 540_2024_3329_MOESM1_ESM.docx]

Supplemental Table

**Patient characteristics:** age, hight, weight, ASA-PS, gestational week, parity (nulliparous or multiparous), fetal growth restriction, co-existing diseases; diabetes mellitus; hypertensive disorders of pregnancy; cardiac disease; thyroid disease; neurological disease; coagulopathy.

**Surgical and postoperative managements:** indication of cesarean delivery (previous cesarean section, fetal presentation, multiple gestation, placental abnormalities, uterine scar, other), type of neuraxial anesthesia (spinal, epidural, CSEA), surgical time, estimated blood loss, intraoperative urine output, total amount of intraoperative fluid, type of fluid (crystalloid or colloid), transfusion, modality of oxytocin administration (bolus with infusion by syringe pump or added into the fluid bag), oxytocin (intraoperative, postoperative 24hrs), methylergometrine (intraoperative, postoperative 24hrs), tranexamic acid (intraoperative, postoperative 24hrs), hypotension after childbirth (defined systolic blood pressure less than 90 mmHg), any notable ECG changes after childbirth, any notable nausea and vomiting after childbirth, requirement of uterine tamponade, vaginal bleeding after surgery.

**Laboratory tests:** preoperative and postoperative hemoglobin, preoperative platelet count, preoperative ATPP, preoperative PT.

ASA-PS: American Society of Anesthesiologists physical status, CSEA: combined spinal epidural anesthesia, ECG: electrocardiogram, APTT: activated partial thromboplastin time, PT: prothrombin time.
